# Laparo-Endoscopic Single Site Combined With Hysteroscopy to Diagnose and Treat Robert’s Uterine Malformation: A Case Report

**DOI:** 10.3389/fsurg.2022.926935

**Published:** 2022-06-14

**Authors:** Xin-Yi Hong, Bo Ding, Yang Shen

**Affiliations:** ^1^ Department of Obstetrics and Gynecology, School of Medicine, Southeast University, Nanjing, China; ^2^ Department of Obstetrics and Gynecology, Zhongda Hospital, School of Medicine, Southeast University, Nanjing, China

**Keywords:** müllerian anomalies, laparoscopy, hysteroscopy combined with laparoscopy, uterine malformation, laparo-endoscopic collaborative surgery

## Abstract

Asymmetric septate uterus, commonly known as Robert’s uterus, is an exceedingly rare uterine malformation described for the first time in 1970 by Robert H. Currently, surgery is the therapy of choice for Robert’s uterus, with surgical choices ranging from laparotomy to minimally invasive surgery. In this paper, we reported that a 14-year-old girl with primary dysmenorrhea that gradually worsened three months after menarche had surgery after many imaging evaluations, and that the intraoperative diagnosis was Robert’s uterus. The diagnostic and therapeutic laparo-endoscopic single site(LESS) combined with hysteroscopy surgery for Robert’s uterine abnormality was shown via a step-by-step presentation of the method accompanied by narrated video footage. During the ten-month postoperative follow-up period, the patient had monthly recurrences with normal menstrual volume and no dysmenorrhea, demonstrating that as a minimally invasive treatment, LESS combined with hysteroscopy surgery is a successful methodfor diagnosing and treating this specific malformation.

## Introduction

Septate uterus malformation refers to all instances in which septal fusion is normal but absorption is aberrant (1). Asymmetric septate uterus, also known as Robert’s uterus, is an exceedingly unusual uterine malformation described for the first time in 1970 by Robert H. (2). It has always been characterized as a singular occurrence with no associated anomalies. The following is a summary of this rare abnormality, as outlined in the literature: (1) primary dysmenorrhea; (2) the external uterine shape is normal during laparoscopic examination, which varies from from its appearance during imaging examination; (3) an isolated incidence with no concomitant deformity (3).

Robert’s uterus is difficult to diagnose. Most instances documented in recent years were preoperatively misdiagnosed, and intraoperatively rectified. There is currently no suggestion for surgical techniques.

### Case Description

**Figure 1:**
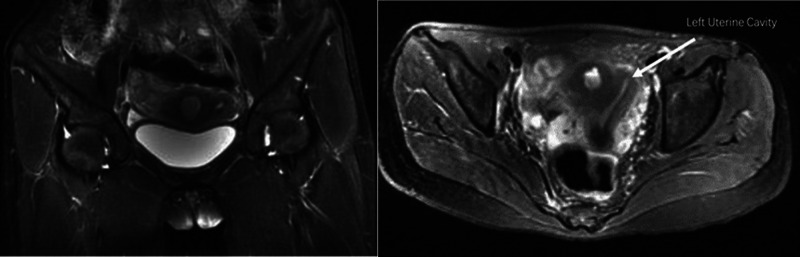
Preoperative pelvic magnetic resonance images.

A 14-year-old girl presented with primary dysmenorrhea that worsened three months after menarche, and magnetic resonance imaging (MRI) showed a probable uterine malformation (Figure 1). A CT urography (CTU) test revealed no evident kidney and ureter abnormalities. Evaluation of external genital organs revealed no evident abnormalities. The hymen was intact while the vagina was unobstructed. Preoperative diagnosis: hemi-uterus (with a functional rudimentary cavity?).

### Diagnostic Assessment

The diagnostic and therapeutic laparo-endoscopic single site (LESS) combined hysteroscopy surgery for Robert’s uterine malformation is described using a step-by-step demonstration of the method with narrated video footage (Supplement video). Before surgery, the patient and her parents had a thorough discussion on the surgical procedure and associated risks were introduced. Consequently, written informed consent of the surgery was obtained from the parents.

**Figure 2:**
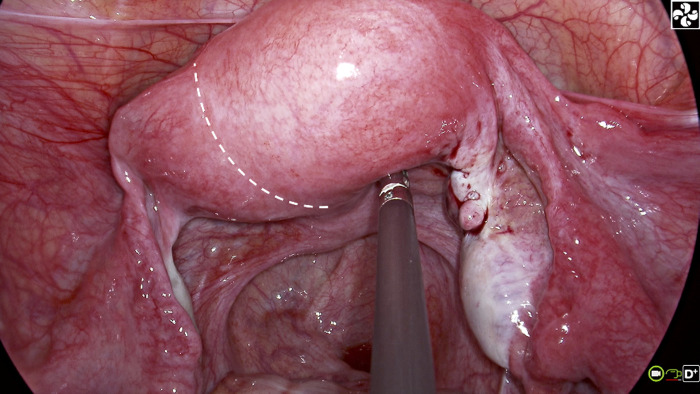
Appearance of uterus during LESS exploration.

Considering the patient’s exam results, a laparo-endoscopic single site (LESS) surgery was arranged. The LESS examination indicated an increased transverse diameter of the uterus and a 0.5 cm concave depression (Figure 2). Asymmetric septate uterus (Robert’s uterus) was diagnosed intraoperatively, and LESS combined hysteroscopic septostomy and uterine fusion surgery was performed. A 5 mm hysteroscopic lens was inserted carefully via the hymen, vagina, and cervix into the left hemi-uterus. The procedure was guided by hysteroscopic video and didn’t need cervix dilation. This procedure took a high degree of surgical ability, but if performed correctly, it would not harm the hymen. The hysteroscopic examination indicated that the uterine endometrium was smooth, and a solitary ostium of the left fallopian tube was visible. After turning off the laparoscopic light, the hysteroscope transmission technique lit just the left hemi-cavity, confirming that the left uterine cavity did not connect with the larger right hemi-uterus cavity. A horizontal incision was made on the fundus to access both sides of the uterine chamber. Cold-knife incision was introduced to protect fertility function and decrease electrothermal injury to the endometrium induced by energy tools. The hysteroscopic lens functioned as a symbolic indicator of the cervix and left uterine cavity, directing the laparoscopic intrauterine septum incision downward towards the endocervix. A COOK guide wire was then retrogradely inserted into the intrauterine cavity, and its tip was retrieved through the vagina. A size 8 Foley catheter with its tip removed, was inserted via the guide wire into the intrauterine cavity and then inflated with 5 mL of normal saline. Considering the future reproductive demands of the patient, the fundus was then doubly sutured. Prescription was made for postoperative estrogen-progesterone cycle therapy to avoid intrauterine adhesion and stimulate endometrial development.

**Figure 3:**
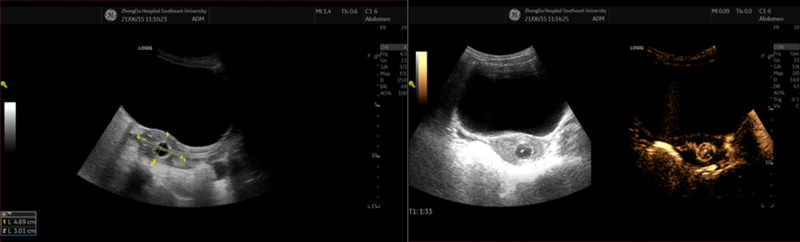
Postoperative ultrasonographic uterine microbubble angiography images (1 week after surgery).

**Figure 4:**
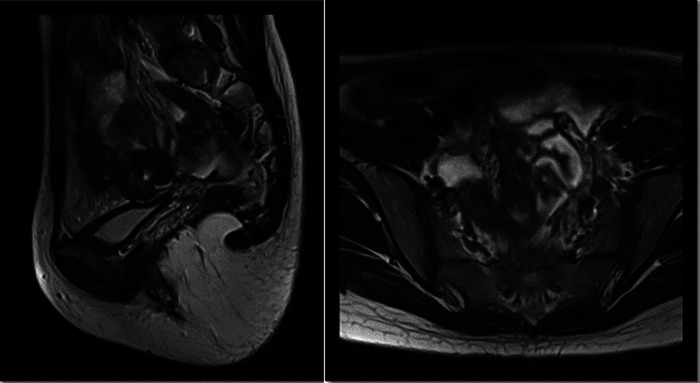
Postoperative pelvic magnetic resonance images (1 month after surgery).

Postoperative ultrasonographic uterine microbubble angiography (Figure 3) and MRI (Figure 4) demonstrated that uterine cavities were connected and the endometrial signals were homogeneous and that the uterine cavities were linked. The patient underwent menstruation about 20 days post-operation. She experienced 10 monthly menstrual recurrences with normal menstrual volume and no dysmenorrhea over the ten months of follow-up.

## Discussion

Robert’s uterus, also known as asymmetric septate uterus, is an extremely rare uterine malformation described for the first time in 1970 by Robert H. (2), belongs to Class U2 of the ESHRE/ESGE classiﬁcation system (Class U2: septate uterus, incorporates all cases with normal fusion and abnormal absorption of the midline septum).

The preoperative diagnosis of Robert’s uterus is difficult and imaging evaluation is required. MRI is the most effective modality for visualizing the uterine septum, the normal external fundal contour, haematometra and haematosalpinx (4). Due to the high quality of tissue characterization, it is regarded as one of the best diagnostic methods for complex anomalies, especially for adolescents and women who have never been sexually active (5) However, 3D ultrasound technology is a viable alternative because it provides highly objective and quantifiable data, is less expensive, and better tolerated by patients. Consequently, it is the ideal perioperative management tool (3). In addition, as an invasive examination, some scholars consider laparoscopy combined with hysteroscopy to be the gold standard for diagnosing Robert’s uterus (5).

The symptoms of Robert’s uterus resemble those of a hemi-uterus with a functional rudimentary cavity and are frequently misdiagnosed. The distinguishing factors are as follows: (1) the hemi-uterine fundus is separated from its functional rudimentary cavity; (2) the depression on the surface of the hemi-uterus and the rudimentary cavity is typically greater than 1 cm; (3) patients with a hemi-uterus have an increased risk of the urinary system (3). Additionally, Robert’s uterus must be distinguished from vaginal oblique septum syndrome and similar conditions.

Currently, surgery is the recommended treatment for Robert’s uterus, with options ranging from laparotomy to minimally invasive surgery (3). Laparotomy is more traumatic but also effective. Additionally, hysteroscopic surgery is a viable alternative. Ludwin et al. used transrectal ultrasound-guided hysteroscopic metroplasty with 4-mm 30-degree optics to treat Robert’s uterine malformation. This technique eliminates the need for abdominal entry and enables precise intraoperative and postoperative assessment of myometrial thickness (6). Nonetheless, the operation has limitations. First, the surgical outcome is not satisfactory. Upon postoperative examination, it was found that the uterine cavity had not returned to its normal shape; thus, a second operation was performed. Second, transrectal ultrasound requires highly skilled surgeons and its proximity to the hysteroscope can impede hysteroscopic procedures (7). LESS combined with hysteroscopy surgery has minimal surgical trauma and effective. In this combined operation, the hysteroscopic lens can be used for both video guidance and auxiliary functions, like a uterine manipulator, and laparoscopy examination can prevent missing pelvic endometriosis, which is extremely common in women with a septate uterus (8). Moreover, according to the Enhanced Recovery After Surgery (ERAS) protocol, minimally invasive surgery is preferred for suitable patients when feasible (9). Consequently, as a LESS procedure, the surgical route conforms this protocol. In identifying and treating uterine malformations, the skill and experience of the laparoscopic surgeon are unquestionably also crucial factors.

In diagnosing and treating Robert’s uterus, postoperative management is also an important procedure. Although it is still debatable whether estrogen can prevent intrauterine adhesions, clinical experience and some studies indicate that the use of estrogen in combination with other ancillary treatments is associated with improved menstrual and fertility outcomes (10, 11). Consequently, postoperative management should currently incorporatehormonal therapy (such as estrogen-progesterone cycle therapy) and ancillary treatments (e.g., intrauterine device, Foley catheter, and hyaluronic acid gel).

If the patient has not yet reached marriage and childbearing age, it is still necessary to continue long-term follow-up to monitor their pregnancy and fertility.

## Conclusion

The management of Robert’s uterus should be appropriate and exact. As a minimally invasive treatment, we have demonstrated that the LESS combined with hysteroscopy surgery is an effective method for the diagnosis and treatment of Robert’s uterine malformation.

## Data Availability

The original contributions presented in the study are included in the article/Supplementary Material, further inquiries can be directed to the corresponding author/s.
